# Phenomenological Analysis of ATP Dependence of Motor Proteins

**DOI:** 10.1371/journal.pone.0032717

**Published:** 2012-03-23

**Authors:** Yunxin Zhang

**Affiliations:** Laboratory of Mathematics for Nonlinear Science, Shanghai Key Laboratory for Contemporary Applied Mathematics, Centre for Computational Systems Biology, School of Mathematical Sciences, Fudan University, Shanghai, China; University of Manchester, United Kingdom

## Abstract

In this study, through phenomenological comparison of the velocity-force data of processive motor proteins, including conventional kinesin, cytoplasmic dynein and myosin V, I found that, the ratio between motor velocities of two different ATP concentrations is almost invariant for any substall, superstall or negative external loads. Therefore, the velocity of motors can be well approximated by a Michaelis-Menten like formula 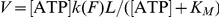, with 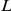 the step size, and 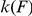 the external load 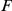 dependent rate of one mechanochemical cycle of motor motion in saturated ATP solution. The difference of Michaelis-Menten constant 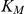 for substall, superstall and negative external load indicates, the configurations at which ATP molecule can bind to motor heads for these three cases might be different, though the expression of 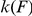 as a function of 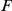 might be unchanged for any external load 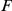. Verifications of this Michaelis-Menten like formula has also been done by fitting to the recent experimental data.

## Introduction

The processive motor proteins, including kinesin, dynein and myosin are essential for biophysical functioning of eukaryotic cells [1], [2]. Due to the development of experimental instrument [3], [4], much accurate experimental data have been obtained [4]–[13]. Both conventional kinesin and cytoplasmic dynein move hand-over-hand along microtubules by converting chemical energy stored in ATP molecules into mechanical works [10], [14]–[17]. Myosin (V or VI) also moves hand-over-hand but along actin filament [8], [18]–[20]. The step size of motor proteins is usually a multiple of their track period. So far, there are many biophysical models to understand the mechanism of motor proteins, including the flashing ratchet model [11], [21], [22], Fokker-Planck equation [23]–[25]. Meanwhile, more detailed mechanochemical models have also been designed to explain the experimental data, and get meaningful biochemical parameters [13], [26]–[31].

In this study, by phenomenological comparison of the velocity-force data of different ATP concentrations, I found that the velocity of processive motor proteins can be described by a Michaelis-Menten like formula 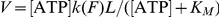, but might with different constant 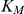 for substall, superstall and negative external loads. The motor velocity in saturated ATP solution is 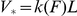, and generally, the velocity of motor can be obtained by multiplying 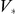 by a constant [ATP]/([ATP]+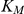).

## Results

**Figure 1:**
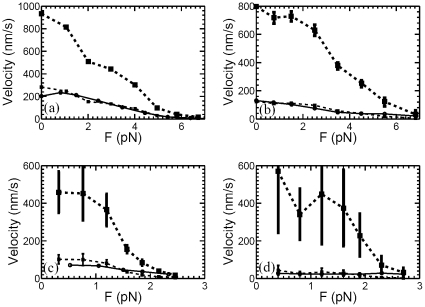
For the positive substall external load cases, the velocity 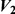 (*solid circles*) of motor proteins at low ATP concentration can be well approximated by the velocity 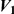 (*big solid squares*) at high ATP concentration divided by a constant (*small solid squares*). (a) For the experimental data of kinesin measured in [6], the velocity 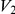 of [ATP] = 10 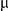
M can be approximated by 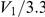 with 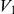 the velocity of [ATP] = 1 mM. (b) For the data of dynein measured in [10], velocity 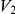 of [ATP] = 10 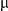
M can be well approximated by 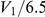 with 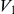 the velocity of [ATP] = 1 mM. (c) For the data of myosin V measured in [8], velocity 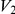 of [ATP] = 10 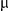
M can be well approximated by 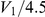 with 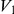 the velocity of [ATP] = 1 mM. (d) For the data of myosin V used in [32] (derived from [5]), velocity 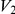 of [ATP] = 1 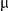
M can be well approximated by 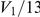 with 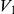 the velocity of [ATP] = 2 mM.

**Figure 2:**
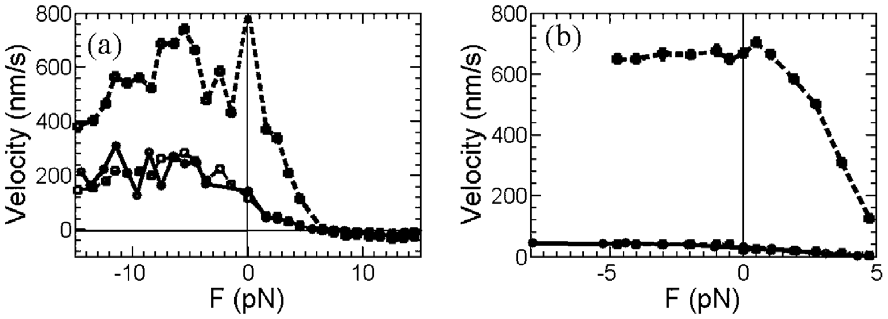
For general external load cases, the velocity 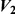 (*solid circles*) of kinesin at low ATP concentration can be well approximated by the velocity 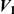 (*big solid squares*) at high ATP concentration divided by a constant (*small solid squares*). (a) For the data in [9], the velocity 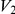 of [ATP] = 10 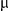
M can be well approximated by velocity 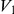 for [ATP] = 1 mM divided by a constant 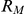 with 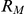 = 2.6 for 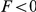, 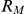 = 7.1 for 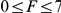 pN, and 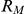 = 2.3 for 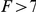 pN. (b) For the data in [7], the velocity 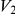 of [ATP] = 4.2 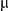
M can be well approximated by velocity 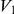 for [ATP] = 1.6 mM divided by a constant 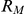 with 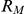 = 16 for 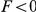, 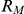 = 29 for 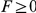.

**Figure 3:**
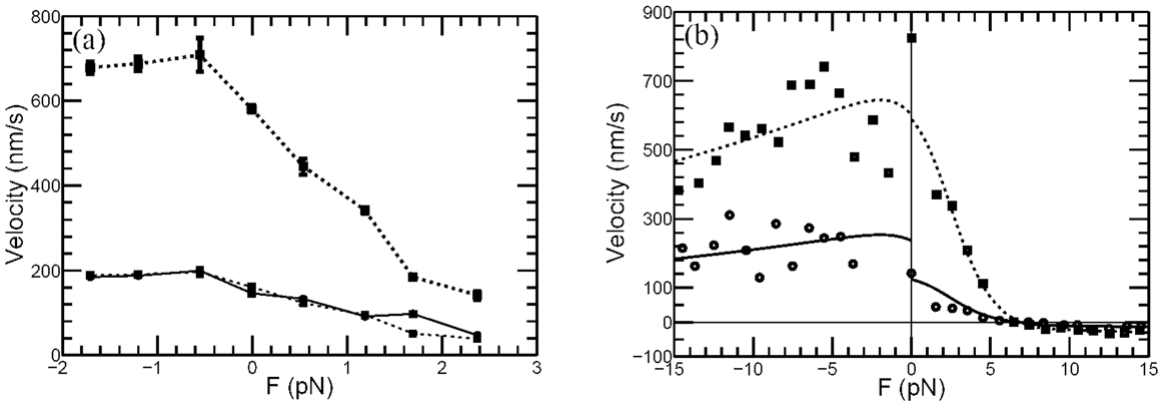
Relation of kinesin velocities at two different ATP concentrations. (a) For the kinesin data measured in [4], the velocity 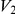 (*solid circles*) of kinesin at low ATP concentration (10 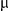
M) can be well approximated by the velocity 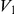 (*big solid squares*) at high ATP concentration (2 mM) divided by a constant 3.6 (*small solid squares*), which is the same for both substall and negative external load. (b) Experimental data for conventional kinesin measured in [9] and the theoretical prediction using the Michaelis-Menten like formula 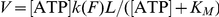. The ATP concentrations are corresponding to [ATP] = 1 mM (dashed line and squares) and 10 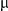
M (solid line and dots) respectively. The model parameter 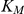 is 15.8 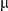
M for 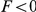, 39.2 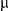
M for 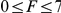 pN, and 11.9 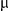
M for 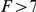 pN, others are listed in Tab. 1.

For the sake of comparison, the velocity-force data of kinesin, dynein and myosin are plotted in Figs. 1, 2 and 3(a). In Fig. 1(a), the thick dashed line 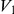 is the velocity-force data of kinesin for [ATP] = 1 mM obtained by Nishiyama *et al* [6], and the solid line 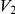 is for [ATP] = 10 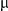
M. One can easily see that there is only little difference between the lines 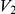 and 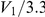. Similar phenomena can also be found for the velocity-force data of dynein and myosin obtained in [8], [10], [32], see Figs. 1(b,c,d). Meanwhile, for negative and superstall force cases, one can find the similar results, but the ratio constants might be different from the positive substall force case, see Figs. 2 and 3(a) for data of kinesin obtained in Refs. [4], [7], [9]. For the kinesin data in [9], the ratio constant is about 2.6 for 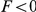, about 7.1 for 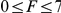 pN, and about 2.3 for 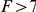 pN [see Fig. 2(a)]. For the data in [7], the ratio constant is about 16 for 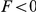, and about 29 for 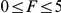 pN [see Fig. 2(b)]. But for the kinesin data measured in [4], the constant 3.6 works well for both substall and negative external load [see Fig. 3(a)].

From the above observations about the experimental data plotted in Figs. 1 and 2, one can see that the velocity-force relation of motor proteins satisfies 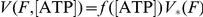. Where 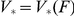 is the velocity-force relation at saturated ATP concentration, and obviously 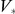 can be written as 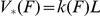 with 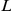 the step size of motor proteins, and 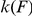 the force dependent rate to complete one ATP hydrolysis cycle (coupled with one mechanical cycle). The function 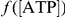 increases with [ATP], 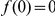 and 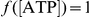 with 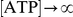. A reasonable form of 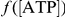 is 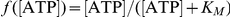 with a parameter 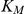 which I called *Michaelis-Menten constant* [7], [33]–[35]. Finally, the velocity formula can be written as 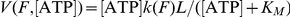.

To verify the above velocity-force formula, the force dependent expression of rate 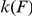 should be given firstly. Usually, the mechanical coupled cycle of ATP hydrolysis includes several internal states, here, as demonstrated in the previous mechanochemical model [27], I assume that, in each cycle, there are two internal states, denoted by state 1 and state 2 respectively.
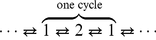
(1)Let 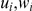 be the forward and backward transition rates at state 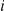, then the steady state rate 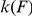 can be obtained as follows [27], [36]

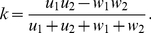
(2)The force dependence of rates 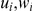 are assumed to be [27]

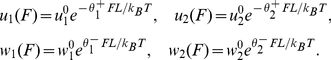
(3)Where 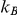 is the Boltzmann constant, 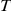 is the absolute temperature, and 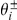 are *load distribution factors* which satisfy 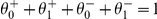. For this two-state model, one can easily get the following formula of motor velocity
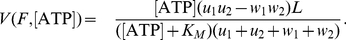
(4)


**Figure 4:**
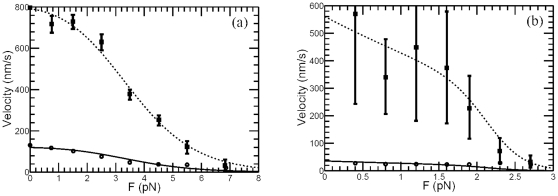
Experimental data for cytoplasmic dynein obtained in [10] and myosin obtained in [5] (see also [32] for the method to get the present values), and the theoretical prediction using the Michaelis-Menten like formula 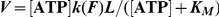. (a) The experimental data are for [ATP] = 1 mM (dashed line and squares) and 10 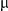
M (solid line and dots). The model parameter 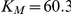 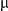
M for 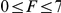 pN. (b) The experimental data are for [ATP] = 2 mM (dashed line and squares) and 1 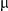
M (solid line and dots). The model parameter 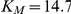 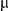
M for 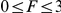 pN.

The fitting results of the above velocity-force formula to kinesin data measured in [9] are plotted in Fig. 3(b). In which, the Michaelis-Menten constant 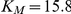 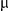
M for 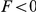, 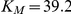 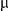
M for 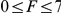 pN, and 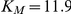 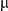
M for 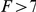 pN, other parameter values are listed in Tab. 1. Meanwhile, the fitting results to the dynein data measured in [10] and myosin data measured in [5] are plotted in Fig. 4(a) and Fig. 4(b) (with Michaelis-Menten constant 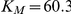 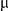
M and 14.8 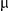
M) respectively, see also Tab. 1 for the corresponding parameter values. The value of 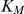 obtained in Figs. 3(b) and 4 might not be consistent with the ratio constant used in Figs. 1 and 2, since the plots in Figs. 1 and 2 are just phenomenological illustration, and the ratio constants are obtained by rough estimation. For example, for the dynein data plotted in Fig. 4(a), 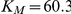 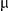
M means the ratio constant between 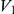 and 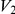 is 6.6, but 6.5 is used in Fig. 1(b). The different values of 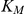 (or 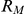 in Fig. 2) for 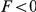, 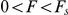 and 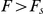 means the possible motor configurations, at which ATP can bind to motor head, might be different for these three force regimes. But in each configuration, the ATP binding rate to motor heads might be the same, i.e. it is independent of the ways used (or time spent) by the motor to get to this configuration. But the time spent by motor proteins to get to such configurations depends on external force 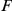. Note, the step size used in the calculations is 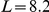 nm for motor proteins kinesin and dynein, but 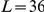 nm for myosin V. Certainly, the same fitting process can also be done to other experimental data. The plots in Figs. 3(b) and 4 indicate that, the experimental data of motor proteins can be well reproduced by the Michaelis-Menten like formula (4), so the phenomenological analysis about the ATP dependence of motor motion is reasonable.

**Table 1:** Parameter values used in the theoretical predictions of the velocity-force relation for conventional kinesin, cytoplasmic dynein and myosin V: see Figs. 3(b) and 4(a)(b).

	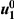	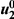	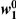	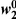	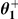			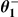	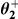	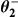
kinesin	716.6	4235.5	0.25			13.5	−0.014	0.609	0.378	0.027
dynein	910.8	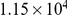	64.0			0	−0.019	0.019	0.386	0.614
myosin	584.0	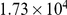	2.55			0	0.03	0.43	0.03	0.51

The unit of rate 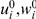 is 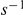.

## Discussion

In summary, in this study, the ATP dependence of motor proteins is phenomenologically discussed. Based on the recent experimental data and numerical calculations, I found the motor velocity can be well described by a Michaelis-Menten like formula 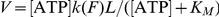 with force dependent rate 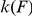 at saturated ATP. The different values of 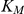 for substall, superstall and negative external load imply, the ATP binding rate to motor heads might be different for these three cases, though the basic mechanism in each mechanochemical cycle (either forward or backward) might be the same. An obvious conclusion from the Michaelis-Menten like formula is that the *stall force*, under which the mean motor velocity is vanished, is independent of ATP concentration [9], [10], [12]. Finally, to describe the ADP concentration dependence of motor velocity, the formula 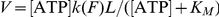 should be changed correspondingly, such as 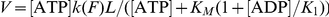 with 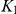 a new parameter [37].
